# Tailored e-Health services for the dementia care setting: a pilot study of ‘eHealthMonitor’

**DOI:** 10.1186/s12911-015-0182-2

**Published:** 2015-07-28

**Authors:** Sandra Schaller, Velislava Marinova-Schmidt, Jasmin Gobin, Manfred Criegee-Rieck, Lena Griebel, Sabine Engel, Veronika Stein, Elmar Graessel, Peter L Kolominsky-Rabas

**Affiliations:** Interdisciplinary Centre for Health Technology Assessment (HTA) and Public Health, Friedrich-Alexander University of Erlangen-Nürnberg, Erlangen, Germany; Chair of Medical Informatics, Friedrich-Alexander University of Erlangen-Nürnberg, Erlangen, Germany; Verein Dreycedern e.V., Specialist unit for informal caregivers in dementia, Erlangen, Germany; Centre for Health Services Research in Medicine, Department of Psychiatry and Psychotherapy, University Hospital Erlangen, Friedrich-Alexander University of Erlangen-Nürnberg, Erlangen, Germany

**Keywords:** e-health, Web portal, Dementia, Caregiver

## Abstract

**Background:**

The European eHealthMonitor project (eHM) developed a user-sensitive and interactive web portal for dementia care: the eHM Dementia Portal (eHM-DP). It aims to provide targeted and personalized support for informal caregivers of people with dementia in a home-based care setting. The objective of the pilot study was to obtain feedback on the eHM-DP from two user perspectives (caregivers and medical professionals), focusing on caregiver empowerment, decision aid, and the perceived benefits of the eHM-DP.

**Methods:**

The study on the eHM-DP was conducted from March 2014 to June 2014. The methodological approach followed a user-participatory design with a total number of 42 participants. The study included caregivers of people with dementia and medical professionals (MPs) from the metropolitan region of Erlangen-Nürnberg (Bavaria, Germany). Study participants were interviewed face-to-face with semi-structured, written interviews.

**Results:**

Caregivers indicated a high degree of perceived support by the eHM-DP and of provided decision aid. In total, 89 % of caregivers and 54 % of MPs would use the eHM-DP if access were provided. The primary benefits participants perceived were the acquisition of individualized information, computerized interaction between caregivers and MPs, empowerment in health-related decisions and comprehensive insights into the progress of the disease. Major recommendations for improving the eHM-DP encompassed: an active search functionality based on predefined terms, the implementation of a chatroom for caregivers, an upload function and alerts for MPs, as well as the overall design.

**Conclusions:**

Our study is the first to have provided new insights and results on an interactive and needs-oriented web portal, endeavouring towards empowerment and assistance in decision making for caregivers as well as MPs within the realm of caring for patients with dementia. The acceptance and willingness to use the eHM-DP emphasizes the potential of eHealth services for community-based dementia care settings.

**Electronic supplementary material:**

The online version of this article (doi:10.1186/s12911-015-0182-2) contains supplementary material, which is available to authorized users.

## Background

The need for care in dementia starts early and increases with alongside the severity of the disease, affecting multiple dimensions such as support for household, financial and social activities, up to nearly constant supervision at severe stages. This implicates a high impact of dementia on people with dementia (PwD), families and healthcare systems [1]. Particularly spouses and children (informal caregivers) provide extensive care, while facing different challenges and, thus, often suffering from additional physical and emotional burdens [2–7]. Therefore, services that target the aid and assistance for informal caregivers are essential. Against this background and due to the projected increase in the number of PwD worldwide [8, 9], there is an urgent need for cost-effective support services.

In the context of aging societies, several studies have highlighted the potential of e-Health services, due to more readily available internet access and the benefits of flexibility, facilitated accessibility and personalized service [10–14]. However, a sustainable implementation of e-Health technologies requires including all the stakeholders that are involved. A lack of user involvement often results in usability problems and higher attrition rates [16]. Thus, frameworks for e-Health technologies emphasize the importance of continuous and systematic evaluations of e-Health services from the user’s perspective early in the development process [16–18]. eHealthMonitor (eHM)’s pilot study aims to involve all users early in the software development process to enable a user-friendly and suitable design. The overall aim of eHM is to provide individualized, personal health knowledge relevant to dementia stakeholders, accompanied with an improvement in the quality and acceptance of electronic healthcare services. Interactive e-Health support services for caregivers have yet to become widely used in real-life healthcare situations in the dementia care setting. Based on the identified user needs, a result of a carefully and comprehensively determining the necessary requirements capture (which has been emphasized in previous studies [10, 12, 18–21]), the eHM Dementia Portal (eHM-DP) was developed for the home-based dementia care setting [22]. To our knowledge, our study is the first of a personalized and interactive web portal, aiming at empowerment and decision aid while considering the perspectives of caregivers and MPs. Currently, the majority of internet-based, supportive interventions for caregivers in dementia are websites or specific educational programs. A minority of evaluated and published studies were similar to the eHM-DP with respect to a combination of information support and interaction functionalities [23–25]. Further online tools such as offered by the Alzheimer’s Association (e.g. Caregiver Stress Check [26]) aim to help find answers, local resources and support, however do not provide interaction with a medical professional. In overall comparison, the eHM-DP differed from previous e-Health service solutions for caregivers in in the combination of seven major respects as requested by users: (1) an interactive and personalized portal with a personal account, (2) computerized communication between MPs and caregivers, (3) tailored support services according to user-specific data in caregiving diaries, (4) a focus on caregiver empowerment and decision aid, (5) addressing the role of MPs, (6) providing individual & longitudinal data on the home-based care setting and the course of the disease (symptoms, medication, well-being), (7) provision of individual & longitudinal data on caregiving tasks and the caregivers’ health status. A further strength of the eHM-DP is its inclusion of a medical professional who is able to receive information on both – the caregiver’s and PwD’s healthcare parameters. While the ‘caregiving diary’ feature focuses on the caregiver’s well-being and living situation, the ‘course-of-disease diary’ and the ‘medication diary’ target the progression of the disease, the overall health status and the safety of PwD.

The purpose of our pilot study is to obtain feedback on the eHM-DP for caregivers of PwD and MPs early in the eHM-DP development process, focusing on empowerment, decision aid, perceived benefits, most promising functionalities as well as recommendations for further improvement.

## Methods

### The eHealthMonitor Dementia Portal functionalities (eHM-DP)

The architecture of the eHM-DP can be divided in three different layers (see Additional file 1): The Presentation layer, represented by the Web Portal, is the front-end of the platform. The eHealthMonitor end-users will exploit the platform by directly interacting with this layer. The Smart layer, composed of the Knowledge on demand subsystem, the Semantic subsystem and the Multi-agent subsystem, are the core components of the platform. The communication and the integration of these three modules enables the provision of personalized and dynamic health recommendations delivered to the eHealthMonitor stakeholders through the above-mentioned presentation layer.

The Data Layer is composed of the external services providing medical and environmental information to the upper layers. The technical design of the eHM-DP was brought forth by means of a service-oriented architecture (SOA) based on the open-source web platform Liferay, modelling and semantic knowledge engineering and multi-agent systems (MAS) [27, 28]. In brief, the available knowledge and data sources are first identified and described using modelling toolkits. Afterwards ontologies are used to semantically lift and integrate the available data by resolving syntactic, structural, and semantic heterogeneities. Finally, agent systems are used to resolve different privacy requirements and conflicting interests, and to reason on existing data. The process of information integration and maintenance of knowledge is semi-automatic and two-tiered. In a first stage agents review periodically the content of websites identified as relevant source of information. In case of updated information compared to last review, domain experts analyze this update and decide about need of incorporation in eHM-DP. The technology used is similar to a mashup. [27] and [28] provide further information on insights into the technological infrastructure of the eHM-DP.

**Fig. 1:**
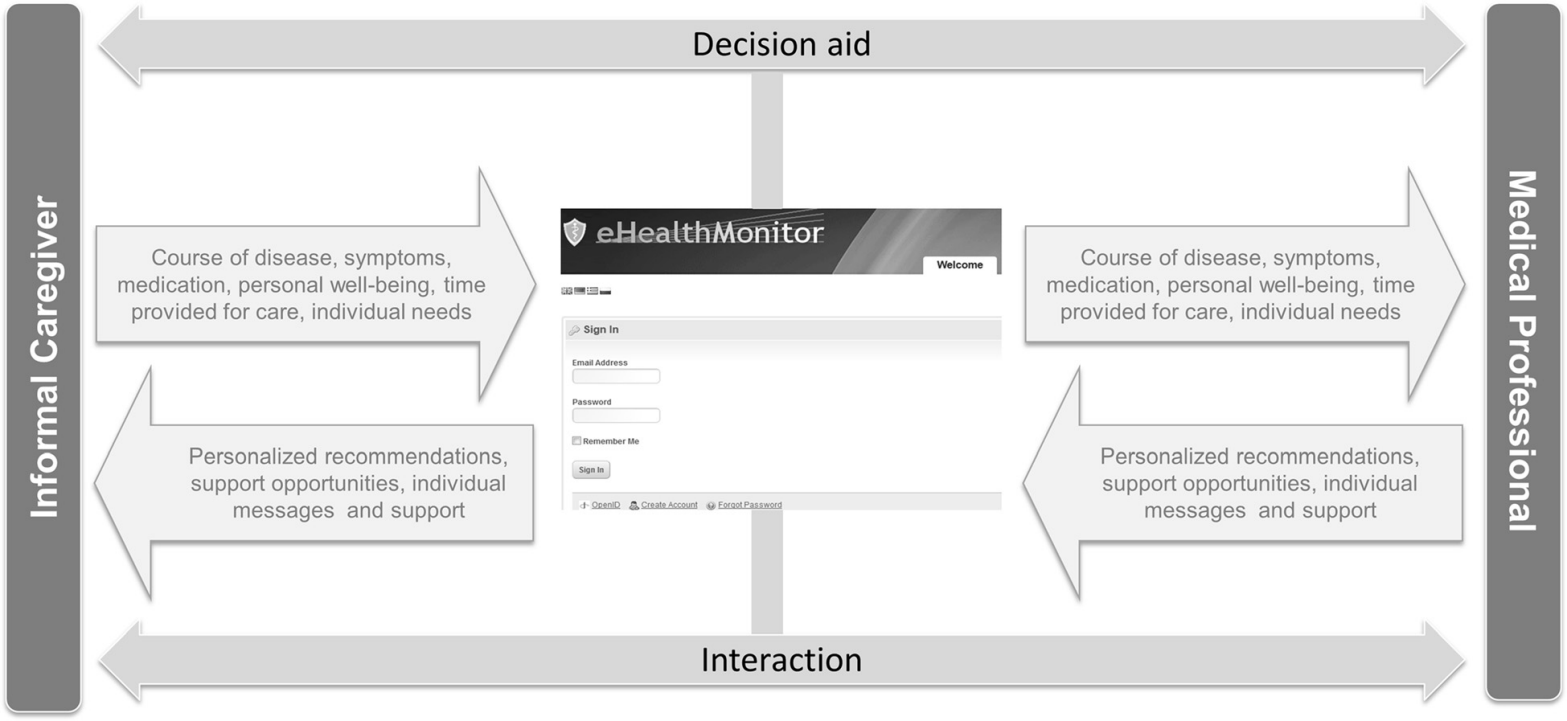
Illustrative representation of the eHM-DP

Based on the aforementioned technologies and rapid and iterative design process between technical and medical partners, caregivers’ needs were integrated based on a) a caregiver focus group, b) interviews with experts in the field and c) reviews of current scientific and evidence-based literature. User access to the eHM-DP was provided via a customizable personal account, thus enabling individualized support services by means of a user-specific profile and user-specific diary entries as quantitative instruments. The eHM-DP is personalized and interactive (Fig. 1), focusing on:

1. An interactive, situation-specific and individualized provision of information and knowledge

Based on the individual registration profiles as well as the electronic diary entries of caregivers (caregiving diary, course-of-disease diary, medication diary), the portal provides individualized, timely and situation-specific information. Information results consist of existing and approved information/guidelines and existing infrastructures (e.g. factsheets by the German Alzheimer Association, Ministry of Health, or local dementia institutions/groups) and of proposed recommendations as communicated by MPs via a messaging feature within the eHM-DP.

(2) Communication with domain experts on dementia

The eHM-DP aims at facilitating and enabling close communication and interaction between caregivers and MPs, striving towards minimizing any burdens caregivers may experience while improving the PwD’s quality of life. Based on individual diary entries (symptoms, well-being, caregiving, medication) as well as specific questions (free text answers), MPs are informed by the eHM-DP (e.g. alerts) and able to provide support via the portal (messaging feature) or directly (appointment, telephone) (Fig. 1).

### Study design

The pilot study on eHM-DP was conducted in the metropolitan region of Erlangen-Nuremberg (Germany). A research design with active user participation based on semi-structured, written interviews was applied to investigate the user’s various attitudes towards and impressions of the eHM-DP. The stimulus within our pilot study was an extensive demonstration of the pilot eHM-DP and user-specific functionalities. The eHM-DP was semi-functional with realization of pivotal benefiting software features. After an introduction, the eHM-DP was accessed by the presenter, using a dummy account and dummy data representing an informal caregiver. Based on the individual profile, the eHM-DP functionalities were demonstrated and explained to informal caregivers. For MPs, the eHM-DP was demonstrated using an account of a medical doctor (dummy data). During the demonstration, questions from study participants were allowed and explanations given. Each demonstration lasted on average 60 to 90 min. Afterwards, the semi-structured interviews were conducted by trained researchers. The interviews were developed according to our research questions and included information on 1) caregiver needs, 2) empowering caregivers, 3) medical decision aid and 4) perceived benefits or recommendations and 5) socio-demographic data (Additional file 2 provides an overview of the main items of the semi-structured interview for participants). Each interview lasted approximately 45 min.

Based on the interview guide developed for the purpose of the study, all interviewers were trained at specific workshops before the study. The pilot study was conducted from 01/03/2014 to 30/06/2014 and approved by the local ethics committee of University of Erlangen-Nürnberg’s Medical Faculty, (Germany). All participants were informed of the objectives and the scope of the study, and provided their informed consent for participation. Data collection and analysis was conducted with exclusively anonymized data. Convenience samples were gathered throughout the development process: caregivers were recruited from a) the Memory Clinic at the University Hospital of Erlangen and b) three caregiver support institutions from the metropolitan region of Erlangen-Nuremberg in Germany. Eligibility criteria for caregivers included: a) primarily responsible, as an informal caregiver, for a person with dementia (according to the International Statistical Classification of Diseases and Related Health Problems, Version 10 (ICD-10): F00 ‘Dementia in Alzheimer disease’; F01: ‘Vascular dementia’; F02: ‘Dementia in other diseases classified elsewhere’ and F03: ‘Unspecified dementia’) living at home, b) at least 18 years of age and c) able to speak, read, and write German. We defined informal caregivers as individuals who provide regular care to a closely related person in need of help for a long period of time, and who did not choose caregiving as an occupation (according to Graessel et al. [29]). Table 1 provides an overview of the caregivers who participated in the study. In addition, eleven MPs were recruited from the Memory Clinic at the University Hospital of Erlangen, caregiver support institutions and day-care/nursing home institutions. Eligibility criteria for MPs included: a) qualified personal in dementia treatment and/or care, b) residing in the metropolitan region of Erlangen-Nuremberg, c) at least 18 years of age and d) able to speak, read, and write German.

**Table 1:** Informal caregiver characteristics

No.	Sex	Age	Relationship to PwD	Residential area	Living together with PwD	Professional status	Support due to cognitive impairment (years)
01	M	61	Child	R	Yes	Retired	8
02	F	83	Spouse	R	Yes	Retired	5
03	F	66	Spouse	R	Yes	Retired	-
04	F	52	Relative	R	Yes	Part-time employed	2
05	F	64	Relative	R	Yes	Retired	2
06	F	31	Relative	U	No	Part-time employed	1
07	F	71	Spouse	U	Yes	Retired	2
08	F	71	Spouse	U	Yes	Retired	4
09	F	68	Spouse	U	Yes	Retired	2
10	F	76	Spouse	U	Yes	Retired	3
11	M	74	Relative	U	No	Retired	8
12	F	51	Child	U	No	Full-time employed	1
13	F	55	Friend	U	No	Homemaker	1
14	F	66	Friend	U	No	Retired	3
15	F	52	Friend	U	No	Homemaker	1
16	F	47	Friend	U	-	Part-time employed	-
17	F	54	Child	U	No	Part-time employed	5
18	F	74	Spouse	U	Yes	Retired	7
19	F	59	Child	R	Yes	Full-time employed	3
20	F	25	Relative	U	Yes	Full-time employed	3
21	F	41	Child	R	-	Part-time employed	-
22	M	29	Relative	R	-	Part-time employed	-
23	F	-	Relative	U	No	Part-time employed	1
24	M	66	Spouse	R	Yes	Retired	1
25	F	70	Spouse	U	No	Retired	Less than 1 year
26	F	32	Relative	U	No	Part-time employed	1
27	M	50	Child	R	No	Unemployed	-
28	M	56	Spouse	R	Yes	Full-time employed	1
29	F	67	Child	U	Yes	Retired	3
30	F	53	Relative	U	No	Full-time employed	2
31	M	75	Spouse	R	Yes	Retired	10
Mean (+/− SD)		58 (+/−15.1)					3.2 (+/−2.6)

*F* female, *M* male, *R* rural area, *U* urban area

### Data analysis

Data collection and analysis was conducted with exclusively anonymized data. The paper-based, semi-structured interviews were captured electronically using SPSS Data Collection Data Entry 7.0. Afterwards, participants were separated into two user groups: Caregivers and MPs. Two researchers structured the data by inductive category development according to Mayring (2000) [30]. The ‘summary content analysis’ technique was applied, in order to reduce the material to core content or aspects. Thereby, steps paraphrasing, generalization to the required level of abstraction, first reduction, second reduction [30] were applied. Descriptive analysis methods were applied using SPSS Statistics 21.0 software.

## Results

A total of 42 participants (31 caregivers; 11 MPs) took part in the pilot study. 46 % of the MPs came from a caregiver support institution (caregiver counsellors; n = 5), 27 % from the Memory Clinic (physicians; n = 3) and 27 % from a day care institution/nursing home (professional caregivers; n = 3). The mean age was 43 years (SD = 12.5; min = 25; max = 58) and almost two thirds (64 %) were female. Furthermore, 31 caregivers, 25 to 83 years old, participated in the study. The mean age was 58 years (SD = 15.1) and over two thirds (77 %) of the caregivers were female. The caregivers were primarily spouses (36 %) or children (23 %), followed by relatives (23 %) and friends (13 %). The health information source ‘internet’ was rated ‘very relevant’ by 43 % of the caregivers and as ‘relevant’ by 23 %, whereas a further 23 % had chosen ‘undecided’ with the remaining 10 % choosing ‘less/not important’. In total 87 % of the caregivers indicated having used the internet for obtaining health-related information. More detailed information on the informal caregivers is provided in Table 1.

### Informal caregiver needs and empowerment

Caregivers indicated a high degree of perceived, individual support from the eHM-DP (average mean = 2.2; SD = 0.9 on a 5-point Likert Scale from 1=’I totally agree’ to 5=’I disagree’; [31]). The provision of individualized information on dementia treatment, local support services, and strategies for preventing caregiver burdens were perceived as the most useful functionalities (Table 2). In addition, the majority of the informal caregivers expressed the need for an active-search functionality based on pre-defined terms. The category ‘more social contacts’ was perceived as less useful, however the recommendation for a chatroom within the eHM-DP was expressed as useful (n = 3) in this context. Caregivers highlighted several issues that are relevant for the daily use of the eHM-DP: hard-copies and a quantitative summary of diary entries, an emergency hotline, and technical support for those less proficient in using the internet.

**Table 2:** Informal caregivers’ perceived support from the eHM-DP (%)

	1	2	3	4	5
	I totally agree (%)	I agree (%)	Un-decided (%)	I rather disagree (%)	I disagree (%)
Informal caregivers’ perceived support by eHM (in %)					
Knowledge about dementia	22	69	9	0	0
Knowledge about dementia treatment	26	74	0	0	0
Knowledge about (local) support services	39	61	0	0	0
Knowledge about financial aspects	26	57	4	13	0
Knowledge about legal aspects	28	55	17	0	0
Knowledge about communication strategies	17	44	35	0	4
Help in critical situations (problems caused by crisis)	23	35	12	18	12
More time for oneself	12	18	12	41	17
More social contacts	17	22	17	16	28
Knowledge about caregiving aspects (nursing skills)	26	43	22	9	0
Knowledge about prevention of caregiver burden	27	73	0	0	0

Furthermore, the most supportive quality of the eHM-DP related to decision making [32] proved to be ‘preparation for doctor visit’ (87 % consent), ‘elaboration of the pros and cons of each option’ (80 % consent) and ‘identification of questions for the doctor’ (76 % consent). In addition, 63 % of the caregivers reported finding the eHM-DP useful when caring for a PwD.

### Medical decision support for MPs

The highest level of support with the eHM-DP was noted as: ‘recognition that a decision has to be made’ (80 %) and ‘preparation to make a better decision’ (80 %). In total 78 % of the MPs indicated that the eHM-DP provides relevant information that is normally very difficult to access (longitudinal data about the disease, time provided for care, caregiver burden, medication history). In total, 67 % of the MPs reported that the eHM-DP provides relevant information for medication treatment, and 55 % stated that eHM-DP readily contributes to establishing contact with another MP/specialist unit. Half of the MPs answered that the eHM-DP facilitates weighing the pros and cons of each (treatment) option. However, MPs highlighted the need for further, additional functionalities within the eHM-DP: An upload function for documents/instruments, improved design (colors, font size, less text), the inclusion of information on medical history such as taking blood or ECG results, an improved interaction functionality for communication between MPs, the possibility of listing PwD based on priority levels and additional information such as local transportation possibilities.

### Primary benefits and recommendations as perceived by the user

The primary perceived benefits and advantages of the eHM-DP for caregivers were: the acquisition of individualized information based on diary entries (35 %), an improved/enabled interaction with MPs (19 %), and empowerment regarding health-related decisions (19 %), followed by a detailed overview of the course of the disease, provision of local support contacts, financial support, real-time access for support and access from home. Caregivers reacted particularly positive to the degree to which the provided information and support was tailored, thus saving them a great deal of time and facilitating the receipt of support according to their individual situation. Overall, 82 % of the caregivers stated that the eHM-DP is a good concept and 89 % would use the eHM-DP if they had access were provided. Altogether 79 % of the caregivers reported that it would be easy for them to become skillful at using the eHM-DP. The overwhelming vocal concerns about the eHM-DP were privacy and data security (19 %). In addition, high time expenditure to use the portal (13 %) and a lack of any personal contact with MPs (10 %) were mentioned. From the MPs’ perspective, the following perceived benefits of the eHM-DP were indicated: an overview of the PwD’s current living/medical situation in a home-based setting (55 %), improved interaction with caregivers (45 %), an improved use of existing dementia support services (27 %), a beneficial overview of medication (18 %), improved interaction from the MPs involved in the treatment and care of a PwD (18 %), empowerment of caregivers (9 %), an improved quality of life for PwD/caregivers (9 %), improved access to information for caregivers (9 %), improved compliance (9 %), improved preparation for follow-up visits (9 %), the provision of information that is typically difficult to access (9 %). Overall, 54 % of MPs would use the eHM-DP if they had access to it, whereas 44 % of the participants indicated ‘undecided’. Major concerns concerned data security (55 %); irregular use of the eHM-DP (36 %), and increased administrative effort (18 %). Examples of quotes from study participants are provided in Additional file 3.

## Discussion

This article describes the participation and perspective of caregivers and MPs for an individualized, interactive web portal (eHM-DP) early in the development process. To our knowledge, our pilot study is the first of a personalized and interactive web portal, aiming at empowerment and decision aid and including perspectives of caregivers and MPs. Thus our study contributes to scientific research by providing new insights into the development of eHealth solutions in dementia care from two important user perspectives. This is crucial for the further development and uptake of eHealth services in the dementia care setting. Furthermore this is particularly relevant against the backdrop of an aging society and limited healthcare expenditures of health care systems, combined with an increasing number of informal caregivers willing to use internet and mobile electronic devices [10, 15, 32–34].

The findings from our pilot study indicate the potential of the eHM-DP for caregivers with regard to caregiver empowerment (knowledge, decision aid), facilitated access to health care services, and promoting interaction with MPs. Only a minority of participants expressed concerns that the use of the eHM-DP would bring a lack of personal contact with MPs. This result is of great importance, as the eHM-DP was designed to complement and not substitute existing treatment and care activities. However, the threat of technologies, to replace valued, human contact has been reported in previous studies [21] and has to be taken into account carefully when introducing new technologies.

From the caregivers’ perspective, the implemented diary features are helpful in increasing their awareness of symptoms and dementia-related topics. Further, in providing assistance in making particular decisions on a situation-to-situation basis in response to individual diary entries. One of three major factors that contributed to these findings was the quality of providing needs-oriented support with the help of the eHM-DP. The provision of individualized information was rated as the most useful benefit as perceived by caregivers, which corroborates to the findings by van der Roest et al. (2010) [25]. Major reasons for its perceived usefulness are the support for individual care situation (help), a reduction of time in searching for information and support, and real-time support. In addition, the functionality of an active search form for specific terms and institutions was requested by caregivers. A second factor that contributed to our findings was the support provided for the caregivers’ specific, unmet needs. These findings are in line with the findings from Brodaty et al. (2005) [6], where the principal reason for the use of community services was the perceived support for unmet needs. The highest level of agreement for support was perceived for: increased knowledge in dementia treatment, knowledge about (local) support services and knowledge about preventing any burdens that may arise while caring for PwD. By increasing awareness of and knowledge on (local) support services, the eHM-DP contributes to reduce barriers for the utilization of existing local support services. This is of great importance, as the lack of knowledge on existing services and dementia infrastructures contributes to one of the four major reasons for non-use of such services [6]. Also, findings from previous studies highlighted the advisable priority of providing information on dementia services and accessing them [6, 30]. The third factor concerns the interaction between caregivers and MPs via the messaging function within the portal, which caregivers perceived as the second most useful benefit of the eHM-DP. These findings are in line with those from Chiu et al. 2009 [23] where users felt that email communication was useful for expressing individual concerns and receiving immediate support from a medical professional. The information provided and interactivity with MPs are major benefits, particularly for reaching caregivers that are immobile (‘home-bound’ due to health status or a lack of transport, public or otherwise) [6] or isolated (e.g. living in rural areas). Thus, the eHM-DP can lower the threshold of access to health care services by accessing support in the privacy of their own homes (without leaving the PwD alone) and by collecting dementia-specific information and knowledge sources. Additionally, the caregiver can use the eHM-DP whenever she/he is free of caregiving duties. In this context, further research is needed to investigate the cost-effectiveness of the eHM-DP. In addition, the eHM-DP would likely benefit from supplemental interaction between caregivers (chat, forum), which was expressed by caregivers during our pilot study. In this case, the eHM-DP would benefit from an identification of existing local or international forums or chats of high quality, such as offered by the Alzheimer’s Association (‘ALZConnected’ [35]). Further aspects that must be taken into account for future portal development are average frequency of use, time of use as well as technical support.

From the MPs’ perspective, the combination of diary features was perceived as very useful since essential and hard to access information that is relevant for medical treatment and care were duly provided, including: longitudinal data on the course of the disease (cognition, ADL, IADL, disturbing behavior, mood, social behavior), longitudinal data on the circumstances revolving around the home-based care (time provided for care, ADL, IADL, caregiver burden) as well as medication history (67 % of MPs reported that the eHM-DP contains relevant information on medication treatment). MPs reported these as being the primary perceived benefits of the eHM-DP, which could potentially also include a preventive aspect. For example, by the prevention of caregiver burden, the eHM-DP has the potential to lower direct (hospitalization, institutionalization) as well as informal costs of care in the dementia setting, particularly against the backdrop of personal burden of informal care is one of the main purposes for nursing home transfers [36, 37]. However, further research is needed to examine this hypothesis and its cost-effectiveness. A second benefit for MPs concerns the improved interaction with caregivers as well as with other MPs involved in the dementia treatment and care process. Further optimization of the eHM-DP would benefit from institution-specific subgroup analysis (e.g. memory clinic vs. caregiver counselling organization). Synonymous to the caregivers’ perspective, data security constituted one of the major concerns of MPs, too. The dilemmas around privacy and autonomy versus safety were already reported by Powell et al. [21] and its debate is crucial for the uptake of an eHealth service. A further aspect was addressed as an insufficient use of the eHM-DP from both parties (caregivers and MPs). This is an understandable concern, as the effectiveness of the eHM-DP depends on regular portal usage from both user groups. However, both, caregivers and MPs stressed the relevance of the needs-oriented, perceived benefits of the eHM-DP, which is an important precondition for proactive and regular use of the portal [6, 21]. According to recommendations from a previous interview study on technologies for caregivers of PwD [21], the eHM-DP shares the need for addressing the following key aspects to enhance the use of the portal: an active facilitation of the service uptake, the need to address barriers of adoption (e.g. privacy issues, impact on caregiver time, computer skills of users), and to emphasize the complementary character of the portal (no substitute to human contact).

In contrast to existing eHealth tools for informal caregivers of a PwD (e.g. DEM-DISC [25], Alzheimer’s Association Online Tools [26, 35]), the eHM-DP is innovative in providing an individualized and interactive web portal which provides specific and several benefits for both, informal caregivers and medical professionals. However, further eHM-DP development should exploit potential synergy effects between existing, complementary systems, such as Ambient Assisted Living (AAL)-systems (e.g. ALLADIN [38]), self-management systems like SmartAssist2 [39], educational online courses (e.g. Mastery over Dementia [40]) or chatrooms (e.g. ALZConnected [35], ANKER [41]).

### Limitations

Although the findings in our study provided essential and new insights into the impact on e-Health support services for caregivers of PwD and for MPs, there are certain limitations that must be taken into account. The first limitation is reflected in the rather small number of participants. However, the strength of our study is that different user perspectives (caregivers, MPs) were included. The second limitation was that no hands-on practice was executed while demonstrating the functionality and use of the eHM-DP, although a demonstration itself was provided. As outlined in the introduction and methods sections, the aim of our pilot study was to involve the user’s perspective early in the development process of the eHM-DP. However a field trial of the portal at the last stage of the project, including the implementation of the pilot study’s results should provide new insights into the impact of the eHM-DP on day-to-day circumstances of caregivers and MPs.

## Conclusion

Our results indicate that the eHM-DP has the potential to meet a number of demands and needs of caregivers of PwD in a home-based care setting. The perceived benefits and willingness to use the system, combined with an increasing number of adults who use the internet regularly, emphasize the potential of personalized and web-based support services for caregivers. Assistance in decision making and empowering caregivers are essential to lowering and preventing caregiver burden affiliated work and/or stresses. E-health interventions can be an efficient alternative to provide personalized support for caregivers at reduced costs [18]. This is especially true for aging societies and limited expenditures of health care systems. The results from the pilot study will be considered in future portal development and questionnaires for the final field trial. Further, our results provide useful insights into and limitations of future e-Health services and their evaluation in the dementia care context. Upon implementing the reported pilot results into the eHM-DP, further research will focus on a) the cost-effectiveness and benefits of the eHM-DP in real-life settings (at home/at work), b) the perceived benefits for specific user subgroups as well as c) the integration of the eHM-DP into existing healthcare infrastructures.

## Additional files

Additional file 1:
**Architecture of eHealthMonitor.** This figure illustrates the technical architecture of eHealthMonitor. Additional file 2:
**Main items of the semi-structured interview for participants.** Additional file 2 illustrates the core items of the conducted semi-structured interviews with caregivers and MPs. Additional file 3:
**Table of themes in content analysis.** This table shows a table of themes identified in the content analysis of the semi-structured interviews.

## Abbreviations

AAL: Ambient Assisted Living
CNA-D: Carer needs assessment in Dementia
eHM: eHealthMonitor
eHM-DP: eHealthMonitor Dementia Portal
ICD: International Classification of Diseases
MAS: Multiagent system
MAX: Maximum
MIN: Minimum
MP: Medical professional
PeKS: Personal eHealth Knowledge Space
PrepDM: Preparation for Decision Making Scale
PwD: Person with Dementia
RCT: Randomized controlled trial
SD: Standard deviation
