# Supplementary figures and images for: Tailored e-Health services for the dementia care setting: a pilot study of ‘eHealthMonitor’
Source: BMC Med Inform Decis Mak. 2015 Jul 28;15:58. doi: 10.1186/s12911-015-0182-2 (PMC4517387; doi:10.1186/s12911-015-0182-2)

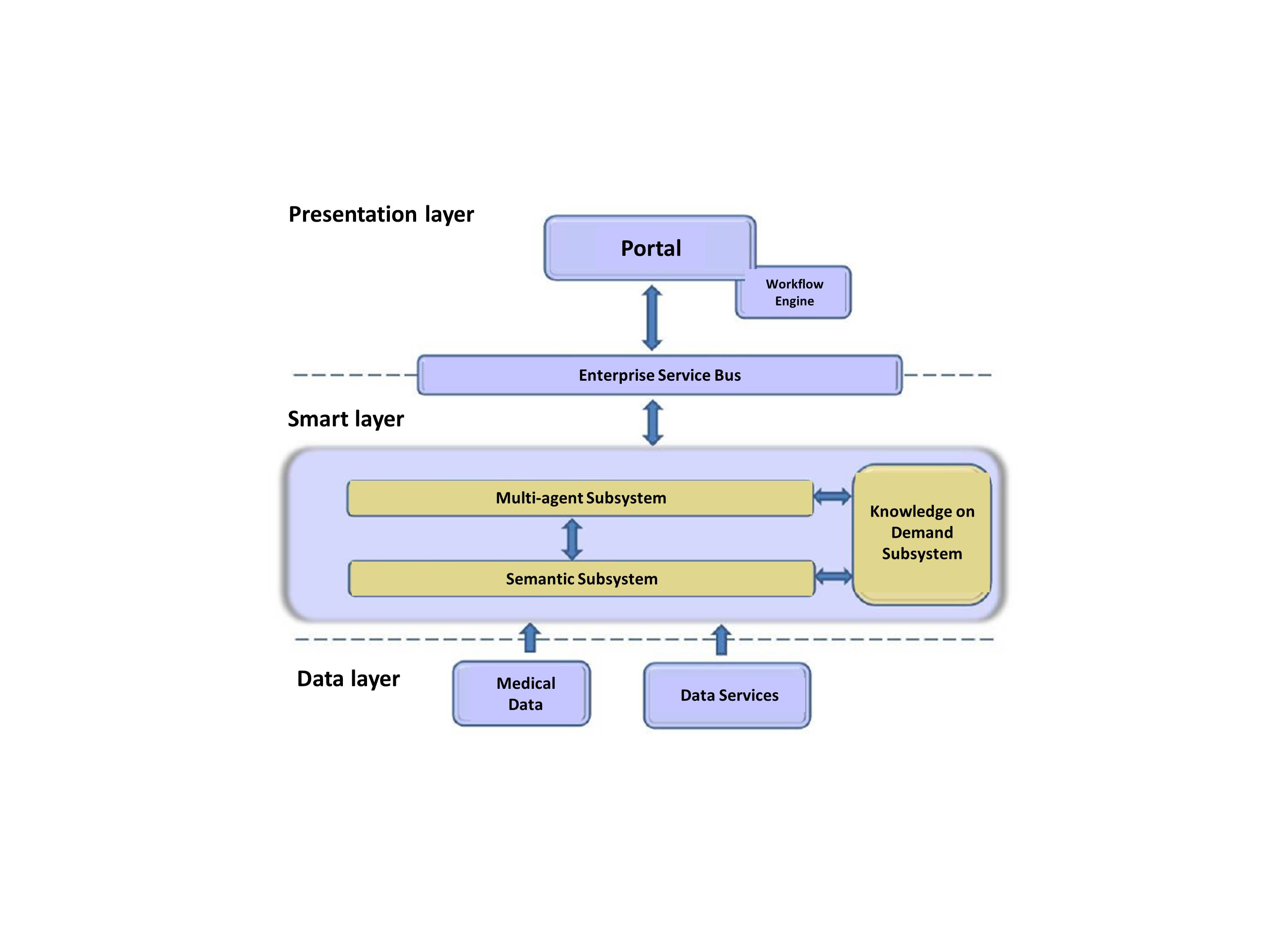

Supplement: Additional file 1: — Architecture of eHealthMonitor. This figure illustrates the technical architecture of eHealthMonitor. [file 12911_2015_182_MOESM1_ESM.jpeg]
