# Supplementary material for: Tailored e-Health services for the dementia care setting: a pilot study of ‘eHealthMonitor’
Source: BMC Med Inform Decis Mak. 2015 Jul 28;15:58. doi: 10.1186/s12911-015-0182-2 (PMC4517387; doi:10.1186/s12911-015-0182-2)
Supplement: Additional file 2: — Main items of the semi-structured interview for participants. Additional file 2 illustrates the core items of the conducted semi-structured interviews with caregivers and MPs. [file 12911_2015_182_MOESM2_ESM.pdf]

Topic	Question	Answers
Information behaviour	How important are the following sources for you to search for health related information (5-point Likert scale)?	Internet<br>TV<br>Books<br>Presentations, courses, workshops<br>Magazines, journals<br>Family, friends, colleagues<br>Pharmacy<br>Direct personal contact in health care context
Needs Assessment / Empowerment	Which needs are addressed by eHM-DP? (Open question)<br>Do you think, the eHM-DP addresses your individual needs as an informal caregiver (pre-selected items based on the CNA-D[26], rated on a 5-point Likert Scale)?	Open answer;<br>CNA-D items (I totally agree (1) to I disagree (5)) [30]:<br>Knowledge about dementia<br>Knowledge about dementia treatment<br>Knowledge about (local) support services<br>Knowledge about financial aspects<br>Knowledge about legal aspects<br>Knowledge about communication strategies<br>Help in critical situations (problems caused by crisis)<br>More time for oneself<br>More social contacts<br>Knowledge about caregiving aspects (nursing skills)<br>Knowledge about prevention of caregiver burden
Decision aid	Do you think, the eHM-DP provides relevant decision support? (Open questions; PrepDM[27], rated on a 5-point Likert Scale)<br>In which situations (and how)? (Open question)	Open answer;<br>Prep-DM items (I totally agree (1) to I disagree (5)) [31]:<br>Help you recognize that a decision has to be made?<br>Prepare you to make a better decision?<br>Help you to think about the pros and cons of each option?<br>Help you to know that the decisions depend of what matters most to you?<br>Help you organize your own thoughts about the decision?<br>Help you think about how involved you want to be in this decision<br>Help you identify questions you want to ask your doctor?<br>Prepare you to talk to your doctor what matters most to you?<br>Prepare you for a follow-up visit with your doctor?<br>Information influenced a decision about how to treat an illness or condition.<br>Information resulted in talking to another doctor or health care provider.<br>Information influenced decision to change medication.
Perceived benefits	What are the main perceived benefits of eHM-DP for your personal situation?<br>Do you think that eHM-DP can contribute to facilitate access to health care services for dementia patients and caregivers?	Open answer;<br>Yes/No
Acceptance, Use	Is there a need for further functionalities/improvements within the eHM-DP?<br>What are your major concerns?<br>I think that eHM-DP is a good concept.<br>If I had access, I would use the eHM-DP.	Open answer;<br>Yes/No
