# Supplementary material for: Tailored e-Health services for the dementia care setting: a pilot study of ‘eHealthMonitor’
Source: BMC Med Inform Decis Mak. 2015 Jul 28;15:58. doi: 10.1186/s12911-015-0182-2 (PMC4517387; doi:10.1186/s12911-015-0182-2)
Supplement: Additional file 3: — Table of themes in content analysis. This table shows a table of themes identified in the content analysis of the semi-structured interviews. [file 12911_2015_182_MOESM3_ESM.pdf]

Theme	User	Quotes from participants (Relevant examples)
**Perceived benefits**		
Aquisition of individualized information	C	-
Empowerment	C	“Direct access to information.”
Overview of the course of the disease	C	“It enables me to see how and when changes occur in the course of the disease.”
Local support contacts	C	“The portal enables a direct access to local support services.”
Financial support	C	-
Access to support from home	C	-
Saving a great deal of time	C	-
Improved interaction with MPs	C, MP	„Very good communication between all stakeholders of the dementia care process (e.g. physicians, professional caregivers, informal caregivers, …).”
History data of the patient (course of disease, medication)	MP	“…especially for elderly patients an overview of history data is very important.”
Improved use of services	MP	“Improved overview about local support services for informal caregivers.”
Improved interaction between MPs	MP	-
Improved quality of life for caregivers and PwD	MP	-
Improved access to information	MP	-
Improved compliance	MP	-
Improved preparation for follow-up visit	MP	-
**Major concerns**		
Time for regular portal use	C	“It is possible, that the usage of the portal will allot a high amount of time. However this is time, the person with dementia itself would benefit, if I spent that time with him instead of with the portal.”
Lack of personal contact to MPs	C	“Portal may replace a personal visit to a physician or a professional caregiver.”
Data security	C, MP	„I have concerns, that the person with dementia feels permanently monitored.”<br>“The data I am entering is very personal data.”
Regular portal use by caregivers	MP	“Interaction depends on regular portal entries from the informal caregiver. The physician needs an alert that interaction is working.”
Increased administrative effort	MP	“There is an increased administrative effort is the system is not integrated into existing IT infrastructures of the clinic.”
**Recommendations for further improvement**		
Active-search functionality based on pre-defined terms	C	-
Chatroom for caregivers	C	-
Hard-copies (e.g. of diaries)	C	-
Quantitative summary of diary entries	C	“Would be good to have an overview about the diaries for the informal caregiver as well.”
Emergency hotline	C	-
Technical support	C	-
Improved design	C, MP	“I would prefer a bigger font and careful color selection especially for elderly people.”
Upload function for documents	MP	„It would be good to include medical records, blood parameter, etc. – so that all MPs are aware of the current medical status and examinations.”
Medical history parameters	MP	-
Improved interaction functionality	MP	“The portal would benefit from an information exchange between all involved medical stakeholders .”
Priority levels	MP	-
Additional information	MP	„
